# The optimization of peritoneal dialysis training in long-term

**DOI:** 10.3389/fneph.2023.1108030

**Published:** 2023-04-06

**Authors:** Meltem Gursu, Larisa Shehaj, Omer Celal Elcioglu, Rumeyza Kazancioglu

**Affiliations:** ^1^ Department of Nephrology, Faculty of Medicine, Bezmialem Vakif University, Istanbul, Türkiye; ^2^ Department of Internal Medicine, Salus Hospital, Tirana, Albania; ^3^ ISN fellow in Department of Nephrology, Faculty of Medicine, Bezmialem Vakif University, Istanbul, Türkiye

**Keywords:** peritoneal dialysis, training, re-training, outcome, peritonitis

## Abstract

Peritoneal dialysis is a home based therapy for patients with advanced chronic kidney disease. This method provides adequate clearance of uremic toxins and removal of excess fluid when a proper dialysis prescription is combined with patient adherence. Peritonitis is the most frequent infectious complication among these patients and may render the continuity of the treatment. Training patients and their caregivers have prime importance to provide proper treatment and prevent complications including infectious ones. The training methods before the onset of treatment are relatively well established. However, patients may break the rules in the long term and tend to take shortcuts. So, retraining may be necessary during follow-up. There are no established guidelines to guide the retraining of PD patients yet. This review tends to summarize data in the literature about retraining programs and also proposes a structured program for this purpose.

## 1 Introduction

Chronic kidney disease is a growing global health issue, with an increasing number of patients requiring kidney replacement therapy. Approximately 11% of patients choose peritoneal dialysis (PD) as their kidney replacement therapy, with this number varying from country to country and center to center (1, 2). Compared to hemodialysis, PD offers several advantages, including fewer hospital visits, fewer hypotension episodes, no anticoagulation requirements, a more independent lifestyle, and greater affordability (3, 4). Peritoneal dialysis is technically simpler to apply (5), minimizes hospital admission and hospital infection (6), is more feasible in rural and remote areas (7), and preserves better the residual kidney function (8, 9) – factors that affect the survival of patients on dialysis (10, 11). Additionally, PD positively affects patients’ quality of life allowing them to maintain employment and daily activities (12). Studies have shown that patients are motivated to select PD because it can be performed independently at night, does not affect their lifestyle, and does not prevent them from traveling (12, 13). However, patients also cite disadvantages of PD therapy, such as catheter care, fear of peritonitis, frequent cycles of bag exchange during the day, lifestyle changes, problems with PD machines, the intensity of cycles, abdominal pain, sleep disturbance and annoyance of other people (14, 15).

Peritonitis is the most critical complication of PD therapy and can lead to a permanent transfer to hemodialysis. Even with effective treatment, it still is associated with mortality (16, 17). The hazard ratio for death due to infection, cardiovascular complications, and noncompliant dialysis is elevated within the first month of a single peritonitis episode and remains raised for up to six months (18). To decrease peritonitis rates, the International Society for Peritoneal Dialysis (ISPD) guidelines recommend preventive measures to decrease peritonitis rates like systemic prophylactic antibiotics application before PD catheter insertion, daily application of antibiotics ointment to the catheter exit site and an itemized training program (19, 20).

Over the past 30 years, the rates of peritonitis have decreased thanks to improvements in connection systems and the use of prophylactic antibiotics before catheter insertion (21). However, despite these advances, the rates still remain high. Reported rates of peritonitis vary greatly between countries, ranging from 0.2 episodes per patient per year (22, 22) to between 0.6 and 0.9 episodes per patient-year (23–25). In addition, the rate differs between different dialysis centers within a country (23, 26, 27). The factors that contribute to this variability are undefined, but it is thought to be due to differences in patient training, infection prevention protocols, and follow-up schedules (28, 29).

Proper training of patients who start treatment with PD is fundamental for achieving a successful technique and a low rate of peritonitis (30). Studies have shown that correct techniques for training patients about PD can significantly reduce the peritonitis rate (31–33). Moreover, re-training programs have been found to be necessary, as peritonitis rates tend to rise over time on PD (34). This may be due to patients becoming more self-confident and neglecting to follow the exact rules taught by medical professionals. Studies analyzing this problem found that improper hand-washing was the most common issue among about 50% of patients, and incorrect mask-wearing was prevalent in around 10-15% of patients (31, 34, 35).

## 2 Exit site care and bag exchanges

The most common factors contributing to the risk of peritonitis are exit site care and connection technique. Patients should be educated on proper techniques and frequently monitored to ensure they are following best practices. Retraining should be provided as necessary. In a randomized controlled study, re-training was shown to lower the rate of exit site infection caused by gram-negative microorganisms per patient per year (36).

According to the 2017 ISPD guidelines, careful consideration should be given to the location of the exit site to ensure it can be easily cleaned and is less prone to trauma (37). After catheter insertion, incisions should be covered and dressing should remain undisturbed for 3-5 days to promote proper epithelialization and wound healing (37). Proper exit site care, including the use of topical antibiotics, avoiding immersion of the peritoneal catheter in water, and immobilization to prevent trauma, plays a crucial role in preventing peritonitis. It is important to follow a sterile dressing technique and wash the exit site with water and antiseptic soap during showers to maintain sterility (37). Exit site cleansing should be performed at least twice a week and after each shower, once the exit site is fully matured (37) Antimicrobial soap and water are commonly used to cleanse the exit site, while povidone iodine and chlorhexidine are common disinfectants (37). Alcohol-based disinfectants should be avoided (37). Although evidence is insufficient to support the use of one solution over another, the guidelines recommend the daily topical application of antibiotic ointments at the exit site to prevent infection (37).

Proper hygienic care including hand washing, use of face masks, and new technologies such as bags with Y set and flush before fill, as well as proper exchange methods are also mandatory to prevent peritonitis in addition to exit site care.

It is important to emphasize the proper performance of bag exchanges during initial training for new PD patients, including checking fingernail cleanliness, bag expiration date and leakage, and avoiding any suspected contamination. Patients should also be instructed on the correct steps to connect and disconnect the bag, flushing before filling, and the importance of wearing a face mask and cap during exchanges. All patients should receive this primary education program covering these issues. The ISPD recommends a training program lasting five days, with each session lasting approximately three hours (20). The VARK learning style questionnaire (Visual, Auditory, Read and write, and Kinesthetic) may be used to facilitate learning (31).

However, a critical question is whether patients apply the taught techniques in the long term. Previous studies have shown that over 50% of patients deviated from the standard procedure, especially in terms of hand washing, during bag exchange in the sixth month of PD, and these errors predicted a higher risk of peritonitis during follow-up (32, 35). To improve adherence, it is important to focus on prevention of infections, signs and symptoms of infections, hydro-electrolytic balance, hand washing, and exchanging/preparing the cycler (32).

Patients may forget the skills they have learned or become overconfident and depart from the standard protocol due to long-term PD treatment (38). Periodic re-training on bag exchange can reduce the risk of contamination by ensuring proper follow-up of the steps of the procedure. However, the guidelines do not specify the content, frequency, or location of the re-training process, as these may vary depending on the characteristics of the patients in each unit.

## 3 The frequency of re-training

The TEACH study compared two groups, frequent re-training (with a home visit every 1-3 months) and conventional re-training (with only two home visits after starting PD), for 24 months. It showed that frequent re-training reduced the risk of exit site infection and peritonitis rate. The study also suggested that older patients are at a higher risk of peritonitis, and repeated training/re-training could benefit this patient subpopulation (39).

The most recent ISPD guidelines recommend re-training after hospitalization, peritonitis, or catheter infection, changes in dexterity, vision, or mental acuity, changes in caregiver for PD exchange, after other interruptions in PD treatment (such as transient transfer to HD), and 3 months after initial training and routinely thereafter (at least once yearly) (40).

## 4 The technique of re-training

A study conducted on pediatric patients found that longer total training time that included theory and practical/technical content was associated with lower peritonitis rates (41). The duration of each training session may also impact the effectiveness of the training. The BRAZPD II study analyzed 2243 incident PD patients from 122 centers in Brazil between 2008 and 2011 and revealed that shorter training sessions (<1 hour/day) and longer total training duration (>15 hours) were associated with lower peritonitis risk (42).

Therefore, extending the total training time with frequent, shorter sessions may allow for the identification and correction of mistakes made during PD therapy implementation.

In a study conducted between December 2010 and June 2016, re-training with technique inspection was compared to oral education. The results showed that repeat training under technique inspection may help correct improper steps during bag exchange and thus reduce the risk of peritonitis (43).

On the other hand, re-training through oral education, which mostly focused on the theoretical part, did not significantly impact the risk of peritonitis, despite patients showing good adherence with the training program (43). Every step of the exchange procedure, including motor skills and memory learning, is stored in the cerebellum and cerebellar cortex (33). This process is more active during technique inspection than it is with oral education. Thus, correcting mistakes during technique inspection can help the patient’s mind store and recall the steps more effectively (33, 44).

## 5 Re-training location

The regular home visits during PD therapy are crucial for follow-up, as both the patient and their family need to receive ongoing support. Early identification and treatment of problems can help keep the patient healthy and reduce hospitalizations. Studies have shown that frequent home visit training can lower the peritonitis rate (45, 46). Since PD is performed in the patient’s home, home visits can provide valuable information on the patient’s environment and how they are carrying out the exchange procedure. Kazancioglu et al. recommended frequent home visits for training, which can help maintain a safe environment and reduce the risk of peritonitis (47).

## 6 Whom to re-train?

Although peritoneal dialysis (PD) is a patient-driven therapy, some patients require assistance from family members or healthcare workers, especially nurses. While the impact of assistance on PD patients is unclear, several observational studies have reported a decreased rate of peritonitis in patients supported by family members or nurses (48, 49). Conversely, studies have shown that patients cared for by private caregivers are more predisposed to peritonitis than those cared for by family members (50, 51).

A study conducted in France between 2000 and 2004 revealed that patients cared for by private nurses had a higher risk of peritonitis compared to those cared for by family members (51). Family members may have a greater personal investment in achieving positive PD outcomes by carrying out the exchange procedures themselves when patients are unable to perform them accurately. When comparing subgroups (family-assisted PD, nurse-assisted PD, and private nurse-assisted PD), family-assisted and nurse-assisted PD had a lower risk of peritonitis (52). Similarly, another study showed that family-assisted PD had a lower risk of peritonitis compared to other groups (53). Therefore, re-training should apply to all participants in the patient’s treatment, including assistants who are healthcare workers.

## 7 The content of the re-training program

We should also consider the content of the re-training program. Should it include all subjects or be limited? In a multicenter study by Ljungman S et al., which included 671 PD patients, all patients received baseline training according to the guidelines (36). Patients were randomized to the re-training group and the control group. Patients in the re-training group performed an exchange with the supervision of a PD nurse without interruption and completed a questionnaire containing 24 multiple-choice questions on hygiene, infection prophylaxis, exchange technique, exit-site infection, and peritonitis. If the patient did not meet the test goals, further training was provided until they were achieved (36). According to the results of the questionnaire, 29% of the patients required re-training. The total incidence of peritonitis and exit-site infection per patient year, as well as outcomes, were similar in both groups. However, peritonitis caused by gram-negative microorganisms was less frequent in the re-training group (36). Although this study was prospective and included a high number of patients, the results were not consistent with previous observational studies. This may be due to differences in patient characteristics, protocols of antibiotic prophylaxis, and compliance with the guidelines. Although there is not enough evidence to follow a certain way, we believe that the content of re-training should be tailored to the patient’s needs.

## 8 Messages from the authors

For both patient and technique survival in PD patients, training of the patients and/or carers is crucial. The training program should be extended throughout the duration of the ongoing PD treatment rather than being restricted to the time when the treatment first began.

While the most recent ISPD guidelines recommended performing re-training three months after initial training and regularly thereafter (once a year at a minimum) and after certain conditions described in the guidelines, we advise closely monitoring the technical capabilities of the patients at the clinic or at home at each visit and performing re-training sessions even more frequently.

Adding more frequent, shorter training sessions may enable health professionals to evaluate patients’ method, identify any deficiencies, and fix them during PD therapy. So, the focus of the retraining session needs to be goal-oriented.

Retraining is applicable in both the hospital and at home. House visits provide useful information regarding the environmental aspects, such as the position of the baths and the cleanliness of the exchange room, which could lead to error during the exchange procedure.

Every participant in the patient’s treatment, notably the patient’s family members, should undergo retraining. The social structure of the family and the community should be considered when making this decision. Even if the assistant is a healthcare professional, retraining should be taken into account.

The re-training program’s content should be adaptable and tailored to the patient’s needs as determined by the PD nurse or the doctor.

Every unit may need to identify the requirements in relation to the patient characteristics.

## Author contributions

All authors contributed equally to collection of knowledge, drafting the manuscript and revision of the manuscript. All authors contributed to the article and approved the submitted version.

## References

[1] Hill NR Fatoba ST Oke JL Hirst JA O’Callaghan CA Lasserson DS. Global prevalence of chronic kidney disease–a systematic review and meta-analysis. PloS One (2016) 11(7):e0158765. doi: 10.1371/journal.pone.0158765 27383068 PMC4934905

[2] Boenink R Astley ME Huijben JA Stel VS Kerschbaum J Ots-Rosenberg M. The ERA registry annual report 2019: Summary and age comparisons. Clin Kidney J (2021) 15(3):452–72. doi: 10.1093/ckj/sfab273 PMC8862051 35211303

[3] Nayak Karopadi A Mason G Rettore E Ronco C. The role of economies of scale in the cost of dialysis across the world: A macroeconomic perspective. Nephrol Dial Transplant. (2014) 29:885–92. doi: 10.1093/ndt/gft528 24516226

[4] Karopadi AN Mason G Rettore E Ronco C. Cost of peritoneal dialysis and haemodialysis across the world. Nephrol Dial Transplant. (2013) 28:2553–69. doi: 10.1093/ndt/gft214 23737482

[5] Mehrotra R Devuyst O Davies SJ Johnson DW. The current state of peritoneal dialysis. J Am Soc Nephrol. (2016) 27:3238–52. doi: 10.1681/ASN.2016010112 PMC5084899 27339663

[6] Ludlow MJ George CR Hawley CM Mathew TH Agar JW Kerr PG. How Australian nephrologists view home dialysis: Results of a national survey: Home dialysis survey. Neph. (2011) 16:446–52. doi: 10.1111/j.1440-1797.2010.01403.x 21518119

[7] Wang V Maciejewski ML Coffman CJ Sanders LL Lee SD Hirth R. Impacts of geographic distance on peritoneal dialysis utilization: Refining models of treatment selection. Health Serv Res (2017) 52:35–55. doi: 10.1111/1475-6773.12489 27060855 PMC5264105

[8] Jansen MA Hart AA Korevaar JC Dekker FW Boeschoten EW Krediet RT. Predictors of the rate of decline of residual renal function in incident dialysis patients. Kidney Int (2002) 62:1046–53. doi: 10.1046/j.1523-1755.2002.00505.x 12164889

[9] Moist LM Port FK Orzol SM Young EW Ostbye T Wolfe RA. Predictors of loss of residual renal function among new dialysis patients. J Am Soc Nephrol. (2000) 11:556–64. doi: 10.1681/ASN.V113556 10703680

[10] Termorshuizen F Korevaar JC Dekker FW van Manen JG Boeschoten EW Krediet RT. Group NS the relative importance of residual renal function compared with peritoneal clearance for patient survival and quality of life: An analysis of the Netherlands cooperative study on the adequacy of dialysis (NECOSAD)-2. Am J Kidney Dis (2003) 41:1293–302. doi: 10.1016/S0272-6386(03)00362-7 12776283

[11] Bargman JM Thorpe KE Churchill DN. CANUSA peritoneal dialysis study group relative contribution of residual renal function and peritoneal clearance to adequacy of dialysis: A reanalysis of the CANUSA study. J Am Soc Nephrol. (2001) 12:2158–62. doi: 10.1681/ASN.V12102158 11562415

[12] Ludlow MJ Lauder LA Mathew TH Hawley CM Fortnum D. Australian Consumer perspectives on dialysis: first national census: National dialysis census. Nephrology. (2012) 17:703–9. doi: 10.1111/j.1440-1797.2012.01651.x 22882456

[13] Dahlerus C Quinn M Messersmith E Lachance L Subramanian L Perry E. Patient perspectives on the choice of dialysis modality: Results from the empowering patients on choices for renal replacement therapy (EPOCH-RRT) study. Am J Kidney Dis (2016) 68(6):901–10. doi: 10.1053/j.ajkd.2016.05.010 27337991

[14] Nakamura-Taira N Muranaka Y Miwa M Kin S Hirai K. Views of Japanese patients on the advantages and disadvantages of hemodialysis and peritoneal dialysis. Int Urol Nephrol. (2013) 45:1145–58. doi: 10.1007/s11255-012-0322-x 23161376

[15] Juergensen E Wuerth D Finkelstein SH Juergensen PH Bekui A Finkelstein FO. Hemodialysis and peritoneal dialysis: Patients’ assessment of their satisfaction with therapy and the impact of the therapy on their lives. Clin J Am Soc Nephrol. (2006) 1:1191–96. doi: 10.2215/CJN.01220406 17699347

[16] Johnson DW Dent H Hawley CM McDonald SP Rosman JB Brown FG. Associations of dialysis modality and infectious mortality in incident dialysis patients in Australia and new Zealand. Am J Kidney Dis (2009) 53(2):290–7. doi: 10.1053/j.ajkd.2008.06.032 18805609

[17] Cho Y Johnson DW. Peritoneal dialysis-related peritonitis: Towards improving evidence, practices, and outcomes. Am J Kidney Dis (2014) 64(2):278–89. doi: 10.1053/j.ajkd.2014.02.025 24751170

[18] Boudville N Kemp A Clayton P Lim W Badve SV Hawley CM. Recent peritonitis associates with mortality among patients treated with peritoneal dialysis. J Am Soc Nephrol. (2012) 23(8):1398–405. doi: 10.1681/ASN.2011121135 PMC3402287 22626818

[19] Segal JH Messana JM. Prevention of peritonitis in peritoneal dialysis. Semin Dial. (2013) 26(4):494–502. doi: 10.1111/sdi.12114 23859192

[20] Li PK Szeto CC Piraino B de Arteaga J Fan S Figueiredo AE. ISPD peritonitis recommendations: 2016 update on prevention and treatment. Perit Dial Int (2016) 36(5):481–508. doi: 10.3747/pdi.2016.00078 27282851 PMC5033625

[21] van Esch S Krediet RT and Struijk DG. 32 years’ experience of peritoneal dialysis-related peritonitis in a university hospital. Perit Dial Int (2014) 34:162–70. doi: 10.3747/pdi.2013.00275 PMC3968101 24584620

[22] Hsieh YP Chang CC Wen YK Chiu PF Yang Y. Predictors of peritonitis and the impact of peritonitis on clinical outcomes of continuous ambulatory peritoneal dialysis patients in Taiwan–10 years’ experience in a single center. Perit Dial Int (2014) 34(1):85–94. doi: 10.3747/pdi.2012.00075 24084840 PMC3923697

[23] Brown MC Simpson K Kerssens JJ Mactier RA. Scottish Renal registry. peritoneal dialysis-associated peritonitis rates and outcomes in a national cohort are not improving in the post-millennium (2000-2007). Perit Dial Int (2011) 31(6):639–50. doi: 10.3747/pdi.2010.00185 21804138

[24] Kofteridis DP Valachis A Perakis K Maraki S Daphnis E Samonis G. Peritoneal dialysis-associated peritonitis: Clinical features and predictors of outcome. Int J Infect Dis (2010) 14(6):489–93. doi: 10.1016/j.ijid.2009.07.016 19926324

[25] Piraino B Bernardini J Brown E Figueiredo A Johnson DW Lye WC. ISPD position statement on reducing the risks of peritoneal dialysis-related infections. Perit Dial Int (2011) 31(6):614–30. doi: 10.3747/pdi.2011.00057 21880990

[26] Davenport A. Peritonitis remains the major clinical complication of peritoneal dialysis: The London, UK, peritonitis audit 2002-2003. Perit Dial Int (2009) 29:297–302. doi: 10.1177/089686080902900314 19458302

[27] Kopriva-Altfahrt G König P Mündle M Prischl F Roob JM Wiesholzer M. Exit-site care in Austrian peritoneal dialysis centers – a nationwide survey. Perit Dial Int (2009) 29(3):330–9. doi: 10.1177/089686080902900319 19458307

[28] Boudville N Johnson DW Zhao J Bieber BA Pisoni RL Piraino B. Regional variation in the treatment and prevention of peritoneal dialysis-related infections in the peritoneal dialysis outcomes and practice patterns study. Nephrol Dial Transplant. (2019) 34(12):2118–26. doi: 10.1093/ndt/gfy204 PMC6887924 30053214

[29] Bender FH Bernardini J and Piraino B. Prevention of infectious complications in peritoneal dialysis: Best demonstrated practices. Kidney Int Suppl (2006) 70:44–54. doi: 10.1038/sj.ki.5001915 17080111

[30] Mawar S Gupta S Mahajan S. Non-compliance to the continuous ambulatory peritoneal dialysis procedure increases the risk of peritonitis. Int Urol Nephrol. (2012) 44:1243–9. doi: 10.1007/s11255-011-0079-7 22102137

[31] Figueiredo AE Bernardini J Bowes E Hiramatsu M Price V Su C. A syllabus for teaching peritoneal dialysis to patients and caregivers. Perit Dial Int (2016) 36:592–605. doi: 10.3747/pdi.2015.00277 26917664 PMC5174866

[32] Russo R Manili L Tiraboschi G Amar K De Luca M Alberghini E. Patient re-training in peritoneal dialysis: Why and when it is needed. Kidney Int Suppl. (2006) 103:127–32. doi: 10.1038/sj.ki.5001929 17080104

[33] Bernardini J. Training and retraining: Impact on peritonitis. Perit Dial Int (2010) 30:434–6. doi: 10.3747/pdi.2009.00244 20628105

[34] Chow KM Szeto CC Law MC Fun Fung JS Li KTP. Infuence of peritoneal dialysis training nurses’ experience on peritonitis rates. Clin J Am Soc Nephrol. (2007) 2:647–52. doi: 10.2215/CJN.03981206 17699477

[35] Dong J Chen Y. Impact of the bag exchange procedure on risk of peritonitis. Perit Dial Int (2010) 30:440–7. doi: 10.3747/pdi.2009.00117 20378843

[36] Ljungman S Jensen JE Paulsen D Petersons A Ots-Rosenberg M Saha H. Peritonitis prevention study (PEPS) trial investigators. retraining for prevention of peritonitis in peritoneal dialysis patients: A randomized controlled trial. Perit Dial Int (2020) 40(2):141–52. doi: 10.1177/0896860819887626 32063220

[37] Szeto CC Li PK Johnson DW Bernardini J Dong J Figueiredo AE. ISPD catheter-related infection recommendations: 2017 update. Perit Dial Int (2017) 37(2):141–54. doi: 10.3747/pdi.2016.00120 28360365

[38] Zhang L Hawley CM Johnson DW. Focus on peritoneal dialysis training: working to decrease peritonitis rates. Nephrol Dial Transplant (2016) 31:214–22. doi: 10.1093/ndt/gfu403 26908816

[39] Chang JH Oh J Park SK Lee J Kim SG Kim SJ. Frequent patient retraining at home reduces the risks of peritoneal dialysis-related infections: A randomised study. Sci Rep (2018) 8(1):12919. doi: 10.1038/s41598-018-30785-z 30150627 PMC6110747

[40] Li PK Chow KM Cho Y Fan S Figueiredo AE Harris T. ISPD peritonitis guideline recommendations: 2022 update on prevention and treatment. Perit Dial Int (2022) 42(2):110–53. doi: 10.1177/08968608221080586 35264029

[41] Holloway M Mujais S Kandert M Warady BA. Pediatric peritoneal dialysis training: characteristics and impact on peritonitis rates. Perit Dial Int (2001) 21(4):401–4. doi: 10.1177/089686080102100412 11587405

[42] Figueiredo AE Moraes TP Bernardini J Poli-de-Figueiredo CE Barretti P Olandoski M. Impact of patient training patterns on peritonitis rates in a large national cohort study. Nephrol Dial Transplant. (2015) 30(1):137–42. doi: 10.1093/ndt/gfu286 25204318

[43] Xu Y Zhang Y Yang B Luo S Yang Z Johnson DW. Prevention of peritoneal dialysis-related peritonitis by regular patient retraining *via* technique inspection or oral education: A randomized controlled trial. Nephrol Dial Transplant. (2020) 35(4):676–86. doi: 10.1093/ndt/gfz238 31821491

[44] Bernardini J Price V Figueiredo A. Peritoneal dialysis patient training, 2006. Peritoneal Dialysis Int (2006) 26(6):625–32. doi: 10.1177/089686080602600602 17047225

[45] Castro MJ Celadilla O Muñoz I Martínez V Mínguez M Auxiliadora Bajo M. Home training experience in peritoneal dialysis patients. EDTNA ERCA J (2002) 28(1):36–9. doi: 10.1111/j.1755-6686.2002.tb00196.x 12035901

[46] Bordin G Casati M Sicolo N Zuccherato N Eduati V. Patient education in peritoneal dialysis: An observational study in Italy. J Ren Care (2007) 33:165–71. doi: 10.1111/j.1755-6686.2007.tb00067.x 18298034

[47] Kazancioglu R Ozturk S Ekiz S Yucel L Dogan S. Can using a questionnaire for assessment of home visits to peritoneal dialysis patients make a difference to the treatment outcome? J Ren Care (2008) 34(2):59–63. doi: 10.1111/j.1755-6686.2008.00023.x 18498569

[48] Xu R Zhuo M Yang Z Dong J. Experiences with assisted peritoneal dialysis in China. Perit Dial Int (2012) 32(1):94–101. doi: 10.3747/pdi.2010.00213 21632447 PMC3525378

[49] Cheng CH Shu KH Chuang YW Huang ST Chou MC Chang HR. Clinical outcome of elderly peritoneal dialysis patients with assisted care in a single medical centre: a 25 year experience. Nephrol (Carlton) (2013) 18(6):468–73. doi: 10.1111/nep.12090 23590458

[50] Hsieh CY Fang JT Yang CW Lai PC Hu SA Chen YM. The impact of type of assistance on characteristics of peritonitis in elderly peritoneal dialysis patients. Int Urol Nephrol. (2010) 42(4):1117–24. doi: 10.1007/s11255-010-9838-0 20848195

[51] Verger C Duman M Durand PY Veniez G Fabre E Ryckelynck JP. Influence of autonomy and type of home assistance on the prevention of peritonitis in assisted automated peritoneal dialysis patients. an analysis of data from the French language peritoneal dialysis registry. Nephrol Dial Transplant. (2007) 22(4):1218–23. doi: 10.1093/ndt/gfl760 17267540

[52] Querido S Branco PQ Costa E Pereira S Gaspar MA Barata JD. Results in assisted peritoneal dialysis: A ten-year experience. Int J Nephrol. (2015) 2015:712539. doi: 10.1155/2015/712539 26600950 PMC4639672

[53] Fang W Ni Z and Qian J. Key factors for a high-quality peritoneal dialysis program–the role of the PD team and continuous quality improvement. Perit Dial Int (2014) 34:35–42. doi: 10.3747/pdi.2013.00120 PMC4076967 24962961

